# Exploring personalized treatment for cardiac graft rejection based on a four-archetype analysis model and bioinformatics analysis

**DOI:** 10.1038/s41598-024-57097-9

**Published:** 2024-03-19

**Authors:** Hongjie Shi, Ming Yuan, Jie Cai, Jiajun Shi, Yang Li, Qiaofeng Qian, Zhe Dong, Gaofeng Pan, Shaoping Zhu, Wei Wang, Jianliang Zhou, Xianwu Zhou, Jinping Liu

**Affiliations:** 1 Department of Cardiovascular Surgery, Zhongnan Hospital of Wuhan University, 169 Donghu Road, Wuhan, 430071 China; 2 Hubei Provincial Engineering Research Center of Minimally Invasive Cardiovascular Surgery, Wuhan, 430071 China; 3 Wuhan Clinical Research Center for Minimally Invasive Treatment of Structural Heart Disease, Wuhan, 430071 China

**Keywords:** Cardiac graft rejection, Co-expression network, Machine learning, Immune cell infiltration, Tyrosine kinase inhibitors, Endomyocardial biopsies, Cardiovascular diseases, Cardiovascular genetics, Genetics

## Abstract

Heart transplantation is the gold standard for treating patients with advanced heart failure. Although improvements in immunosuppressive therapies have significantly reduced the frequency of cardiac graft rejection, the incidences of T cell-mediated rejection (TCMR) and antibody-mediated rejection remain almost unchanged. A four-archetype analysis (4AA) model, developed by Philip F. Halloran, illustrated this problem well. It provided a new dimension to improve the accuracy of diagnoses and an independent system for recalibrating the histology guidelines. However, this model was based on the invasive method of endocardial biopsy, which undoubtedly increased the postoperative risk of heart transplant patients. Currently, little is known regarding the associated genes and specific functions of the different phenotypes. We performed bioinformatics analysis (using machine-learning methods and the WGCNA algorithm) to screen for hub-specific genes related to different phenotypes, based Gene Expression Omnibus accession number GSE124897. More immune cell infiltration was observed with the ABMR, TCMR, and injury phenotypes than with the stable phenotype. Hub-specific genes for each of the four archetypes were verified successfully using an external test set (accession number GSE2596). Logistic-regression models based on TCMR-specific hub genes and common hub genes were constructed with accurate diagnostic utility (area under the curve > 0.95). RELA, NFKB1, and SOX14 were identified as transcription factors important for TCMR/injury phenotypes and common genes, respectively. Additionally, 11 Food and Drug Administration-approved drugs were chosen from the DrugBank Database for each four-archetype model. Tyrosine kinase inhibitors may be a promising new option for transplant rejection treatment. KRAS signaling in cardiac transplant rejection is worth further investigation. Our results showed that heart transplant rejection subtypes can be accurately diagnosed by detecting expression of the corresponding specific genes, thereby enabling precise treatment or medication.

## Introduction

Since the first human heart transplant was performed 50 years ago by Dr. Christiaan Barnard, heart transplantation (HTx) has become the gold standard procedure for treating refractory heart failure^1–3^. According to the 37th Annual Adult Heart Transplant Report by the International Society for Heart and Lung Transplantation (ISHLT) Thoracic Organ Transplant Registry, the number of transplants performed worldwide increased by at least 5% from 2010 to 2018, compared to the average number performed from 1992 to 2000^4^. Cardiac allograft rejection is a major cause of graft damage and mortality^4,5^. Even after immunosuppressive drugs have been administered, the lethality of immune rejection remains high^6^. T cell-mediated rejection (TCMR) and antibody‐mediated rejection (ABMR) are two subtypes of cardiac allograft rejection recognized by the ISHLT that can be diagnosed histologically via endomyocardial biopsy (EMB)^7–9^. Recently, a heart molecular microscope diagnostic (MMDx) system enabling more accurate diagnosis of HTx rejection based on three molecular phenotypes, including stable (normal), TCMR, and ABMR (three-archetype analysis, 3AA)^10^, was successfully constructed by Philip F. Halloran et al. To better distinguish the inflammatory response caused by tissue injury from cardiac allograft rejection, injury was added to the 3AA model, resulting in four-archetype analysis (4AA) of the stable (normal), TCMR, ABMR, and injury phenotypes^11^. However, little is known regarding specific genes and the detailed functions of the different phenotypes. The exploration of these core genes will contribute to the development of specific non-invasive diagnostic methods for different subtypes of heart transplant rejection.

EMB has been routinely used to monitor post-transplant rejection reactions in the heart, but its accuracy is often affected by the experience of the performing physician, sampling variability, and the interpretation by pathologists. As an invasive procedure, it is associated with various complications, including graft injury, making it potentially fatal for transplant patients^12^. AlloMap is presently the sole clinically employed method for monitoring cardiac transplant rejection, which enables accurate monitoring of acute cellular rejection in low-risk individuals within the 6-month to 5-year period following transplantation^13^. The AlloMap assay (CareDx, Inc.; Brisbane, CA) was developed based on the belief that messenger RNA (mRNA) derived from circulating peripheral blood mononuclear cells (PBMCs) can indicate the occurrence of acute cellular rejection earlier than traditional biopsy-based screening methods. This is achieved by analyzing a distinctive gene expression pattern associated with immune system activation and leukocyte movement^14^. However, AlloMap lacks sensitivity and specificity in detecting acute rejection reactions occurring within the first two months after cardiac transplantation, as well as antibody-mediated rejection reactions. To address this limitation, Lee et al. conducted a combined analysis of the MMDx system and AlloMap, identifying a circulating blood gene, ITGA4, with specificity for ABMR^15^. Further clinical cohort studies are warranted to validate these findings. Multiple techniques, including echocardiography^16^, cardiac magnetic resonance imaging (CMR)^17^, nanomaterials^18^ and fluorescent probes^19^, have been explored for the diagnosis of cardiac transplant rejection from various dimensions. However, their clinical application still has a long way to go.

Immunosuppressive therapy is the main treatment method for transplant rejection, aiming to inhibit immune cells and circulating antibodies to suppress immune rejection. Various immunosuppressive agents, including cyclosporine, tacrolimus, sirolimus, and myelosuppressive agents, have been developed and widely used in clinical practice^20^. While immunosuppressive therapy reduces the occurrence of organ rejection, it also increases the risk of infections, kidney damage, and even malignancies in patients. Some patients may develop tolerance to immunosuppressive drugs, resulting in poor treatment outcomes^21^. Traditional immunosuppressive therapy has limited effectiveness against antibody-mediated rejection. Therefore, there is an urgent need for further research and development of novel therapies for cardiac transplant rejection to improve patient survival and quality of life.

Recent studies have found that inhibition of Janus tyrosine kinase 3^22^ and Src kinases^23^ can significantly improve the occurrence of post-cardiac transplant rejection. This suggests that tyrosine kinase inhibitors may be a promising new option for transplant rejection treatment. Tyrosine kinases are enzymes involved in various cellular signaling pathways, including cell proliferation, differentiation, survival, and migration. Overactivation of tyrosine kinases is associated with the development and progression of various diseases, including cancer, inflammatory disorders, and autoimmune diseases^24^. Tyrosine kinase inhibitors block the activity of tyrosine kinases, disrupt abnormal signaling pathways, inhibit immune cell activation, impede immune cell migration, and thus suppress disease progression^25^. Some tyrosine kinase inhibitors have been approved for the treatment of tumors such as breast cancer and chronic myeloid leukemia^26,27^. However, considering that tyrosine kinase inhibitors used in cancer treatment often come with severe cardiovascular toxicities, careful selection of reasonable tyrosine kinase inhibition targets in cardiac transplant rejection is crucial.

In addition, recent research has reported significant inhibition of chronic rejection in cardiac transplantation through targeted intervention of macrophage RAS family proteins^28^. The RAS signaling pathway is an important cellular signaling pathway involved in the regulation of cell proliferation, differentiation, and survival. It is one of the most commonly mutated families in cancer^29^. KRAS is one of the most important proteins in the RAS family, and its activation controls multiple signaling cascades, including the RAF-MEK-ERK pathway, PI3K-AKT-mTOR pathway, and RALGDS-RAL pathway. Activation of these downstream signaling pathways stimulates cell proliferation, migration, and ultimately contributes to tumorigenesis^30^. Several clinically approved RAS protein-related inhibitors have been developed for the treatment of various cancers^31,32^. Whether KRAS signaling is involved in cardiac transplant rejection warrants further investigation.

In this study, we used various algorithms such as the WGCNA, LASSO regression, and SVM-RFE algorithms to identify the TCMR/ABMR/injury-specific genes and common genes based on Gene Expression Omnibus (GEO) accession number GSE124897. One ABMR-specific hub gene (GNLY), one injury-specific hub gene (CSF1R), six TCMR-specific hub genes (including CD8A, HLA-A, CCR7, CD72, ZAP70 and LTB), and eight common hub genes (including ICAM1, CXCXL10, CXCL9, HLA-DPA1, CTSS, TAP1, STAT1 and HLA-DMA) were successfully screened. RELA was considered a negative transcriptional factor for the TCMR-specific gene set and common gene set. NFKB1/SOX14 was selected as a positive transcription factor for the common/injury gene set. Based on the six TCMR-specific hub genes and eight common hub genes, two logistic-regression models were constructed with higher diagnostic utility for the TCMR or rejection phenotypes (area under the curve [AUC] > 0.95). GNLY/CSF1R could be regarded as a potential diagnostic marker for the ABMR/injury subtype (AUC > 0.85). Eleven drugs approved by the Food and Drug Administration (FDA) were identified using the DrugBank Database. In a word, our study identified the specific genes for different cardiac rejection subtypes which can diagnose cardiac graft rejection well. Transcription factors and targeted drugs for different cardiac rejection phenotypes were also predicted. Tyrosine kinase inhibitors and KRAS signaling may be a good research direction for treating heart transplant rejection.

Based on these findings, we expect that different heart transplant subtypes can be accurately identified and treated with specific drugs, which may better mitigate graft loss associated with graft rejection.

## Results

### Identifying gene modules correlating with different heart transplant rejection phenotypes

**Figure 1:**
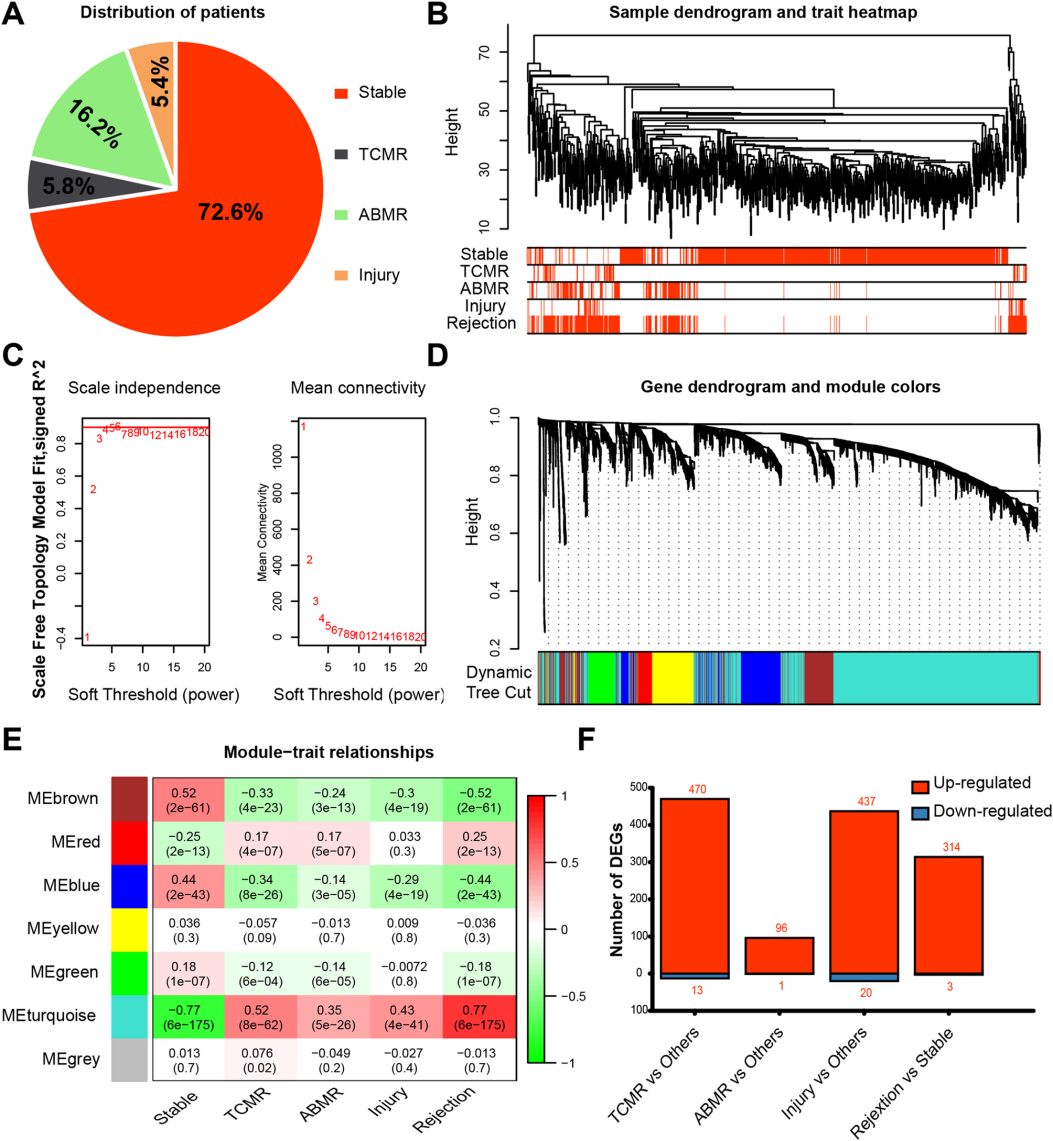
Construction of co-expression network and identification of highly expressed genes in different phenotypes. (**A**) The percentage of stable/TCMR/ABMR/injury samples in GSE124897. (**B**) Sample tree and trait heat map of 889 samples. (**C**) Scale free topology when soft-thresholding power β = 4. (**D**) Gene dendrogram of identified co-expressed genes in modules. The different colors mean different modules. (**E**) A heatmap of module-trait correlation (person correlation analysis). The up number in the matrix cell represents the correlation coefficient and the bottom number represents the p-value. Different colors represent different gene modules. (**F**) Boxplot of the number of DEGs in different groups.

The gene-expression matrix for 889 samples in GSE124897 was imported into the WGCNA algorithm. After calculating the gene variances, the top 25% variant genes (5020 genes) were chosen to construct a co-expression network. Ten outliers were detected and eliminated using a sample-clustering tree (Fig. 1B, Supplementary Fig. 2A). Then, a scale-free network was built based on β = 4 (scale-free R^2^ = 0.9) (Fig. 1C, Supplementary Fig. 2C). Next, an adjacency matrix was generated and converted into a TOM. Finally, seven gene modules were generated according to average-linkage hierarchical clustering, using the TOM-based dissimilarity measure (Fig. 1D). The subtype information was extracted for all 889 samples and linked to the gene modules. A turquoise gene module was identified as a key module based on its significant correlation with the stable (r = − 0.77, p < 0.0001), TCMR (r = 0.52, p < 0.0001), ABMR (r = − 0.35, p < 0.0001), injury (r = 0.43, p < 0.0001), and rejection (r = 0.77, p < 0.0001) phenotypes (Fig. 1E, Supplementary Fig. 2B).

### Identifying highly expressed genes in the ABMR, TCMR and injury samples

To obtain highly expressed genes in the ABMR, TCMR and injury samples, we used the limma R software package to study differences between the TCMR, ABMR, injury, stable, and rejection phenotypes (log2 FC ≥ 1, FDR < 0.05). With the TCMR, ABMR, injury, and rejection samples, we identified 470, 96, 437, and 314 upregulated genes, respectively. In addition, 13, 1, 20, and 3 downregulated genes were found with the TCMR, ABMR, injury, and rejection samples, respectively (Fig. 1F, Supplementary Fig. 2D). Only highly expressed genes were selected for further analyses.

### Identifying immune-related candidate TCMR-, ABMR-, injury-specific genes

**Figure 2:**
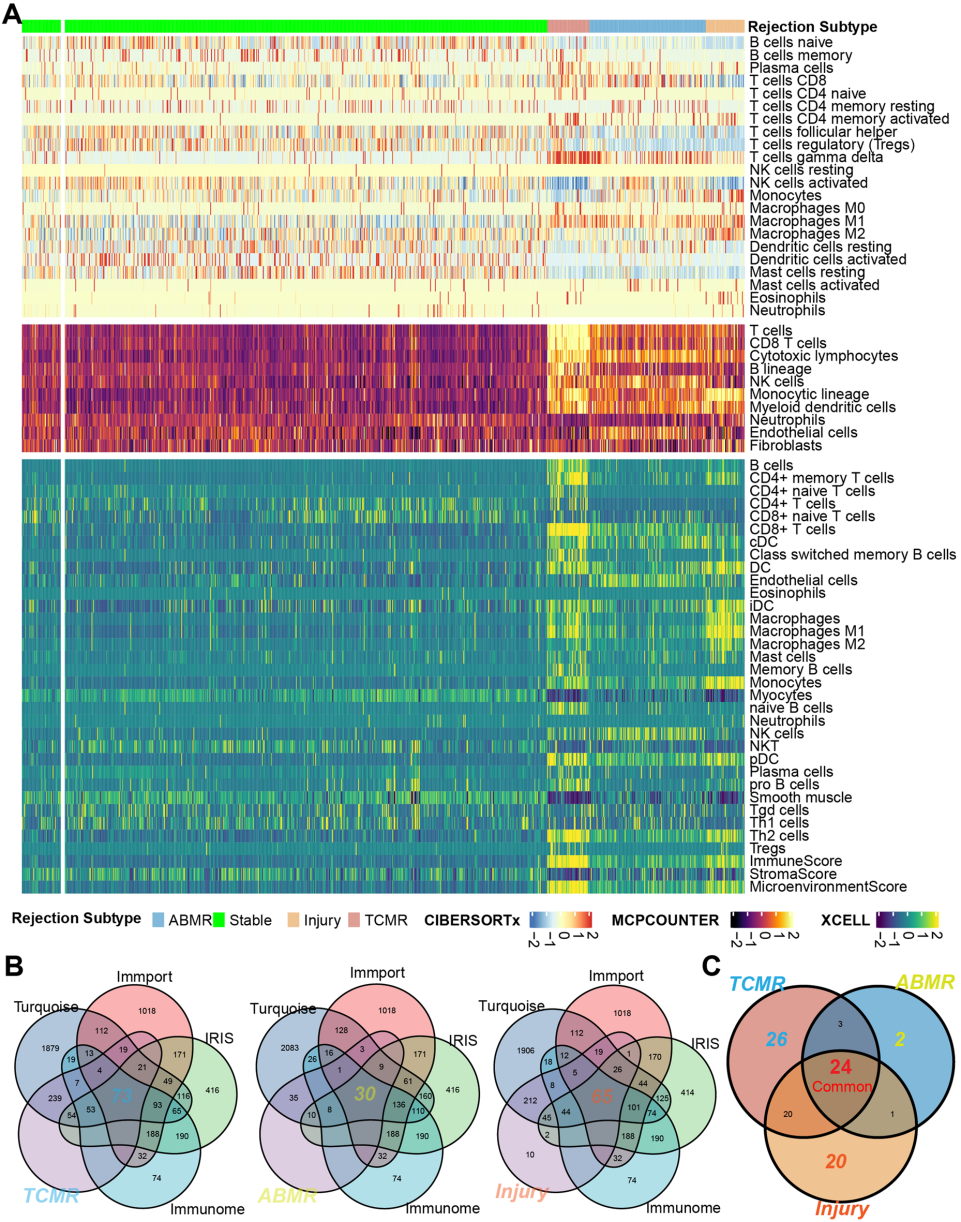
Identification of ABMR/TCMR/injury specific genes. (**A**) Immune landscape for different phenotypes. (**B**) The intersecting genes of turquoise module genes, immune genes markers and highly expressed genes in ABMR/TCMR/injury subtypes were considered to be key genes. (**C**) The intersecting genes of ABMR, TCMR and injury key genes were identified as ABMR/TCMR/injury specific genes.

According to the immune-infiltration heatmap for different phenotypes, generated based on CIBERSORTx, MCPcounter, and X-Cell algorithms, more immune cell infiltration occurred in the ABMR/TCMR/injury samples than in the stable samples (Fig. 2A). Three immune-related gene lists were downloaded from the IRIS Database (1489 genes), the Immport Database (1793 genes), and the Immunome Database (881 genes). The intersecting genes of turquoise-module genes and highly expressed genes in ABMR/TCMR/injury samples present in the three immune-related gene lists, were considered as key genes for the ABMR/TCMR/injury samples (30, 73, and 65 genes, respectively) (Fig. 2B). Finally, by determining the intersection of key ABMR, TCMR, and injury genes, we identified 26 TCMR-specific genes, 2 ABMR-specific genes, 20 injury-specific genes, and 24 shared genes (Fig. 2C).

### Biological function analysis and construction of a protein–protein-interaction (PPI) network

**Figure 3:**
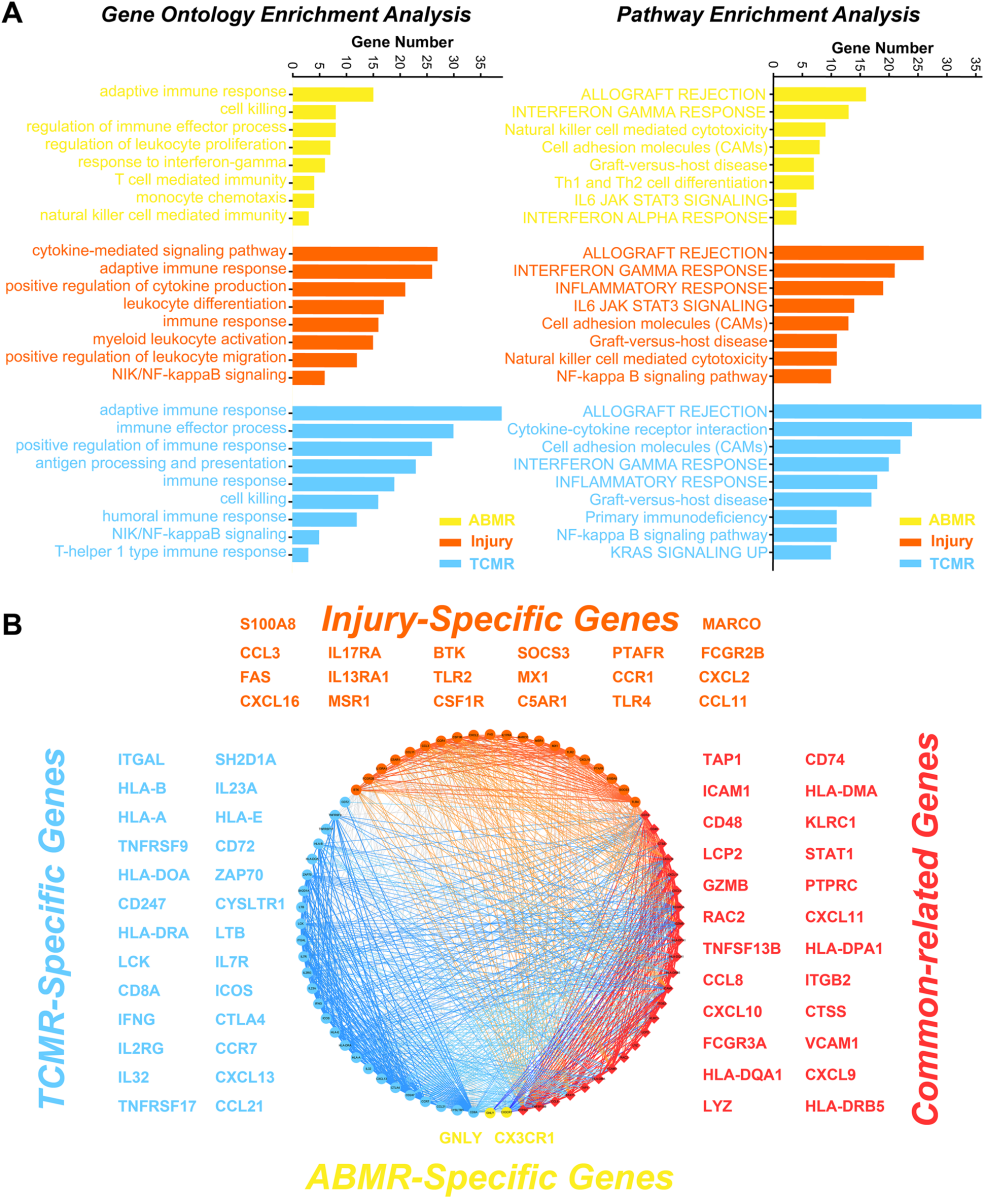
Biological function and PPI network analysis. (**A**) Gene oncology and pathway enrichment analysis based on the ABMR/TCMR/injury key genes. (**B**) PPI network construction using ABMR/TCMR/injury specific genes.

Key ABMR/TCMR/injury genes were imported into Metascape for GO and pathway-enrichment analysis. The top eight GO and enriched pathways are shown in Fig. 3A. In terms of GO analysis, these key genes were mainly enriched for terms related to immune-related functions such as adaptive immune response, cell killing, T cell mediated immunity, and NIK/NF-kappaB signaling. Regarding pathway enrichment, these key genes were significantly enriched for terms related to immune rejection-related pathways such as allograft rejection, graft-versus-host disease, cell adhesion molecules (CAMs), and natural killer cell-mediated cytotoxicity. Strikingly, in addition to these shared biological functions, we found that the key TCMR genes were specifically enriched for T cell-related functions, that key ABMR genes were specifically enriched for B-cell related functions, and that the key injury genes were specifically enriched for chemokine and leukocyte migration-related functions (Supplementary Fig. 3). These results suggested that the selected key ABMR/TCMR/injury genes could serve as specific diagnostic markers for corresponding heart transplant rejection phenotypes. After identifying ABMR-, TCMR-, injury-specific genes, as well as common genes, all genes were input into the STRING Database^33^ to construct a PPI network. The results revealed strong interactions between specific genes associated with different phenotypes (Fig. 3B).

### Predicting TFs for different phenotypes

**Figure 4:**
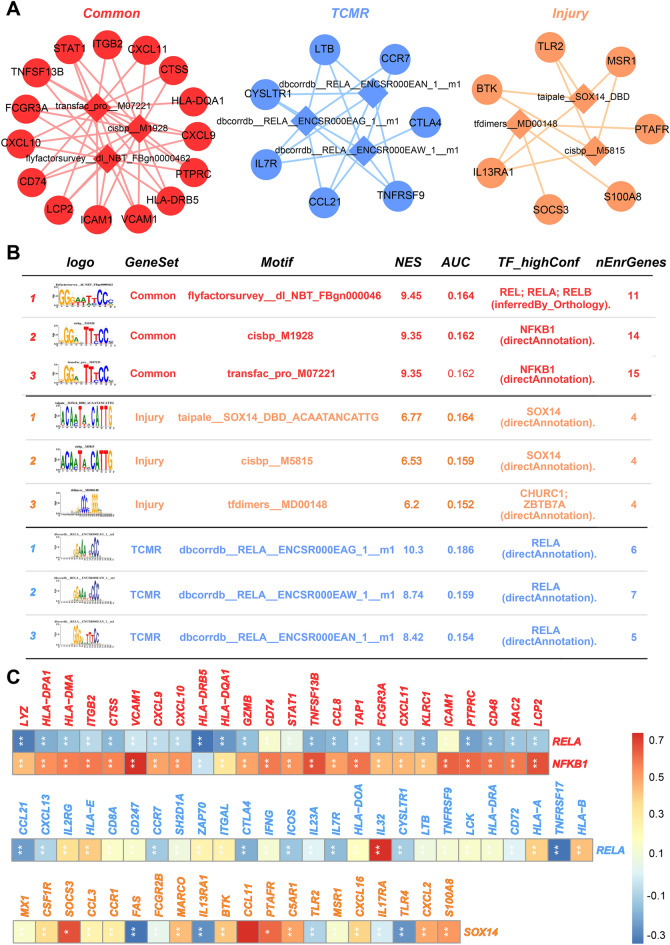
Prediction of transcription factors for different subtypes. (**A**) Top 3 enriched motifs for common genes and TCMR/injury specific genes. The diamonds represent motifs and the circles represent candidate genes. (**B**) Exhibition of top 3 enriched motifs. (**C**) Person correlation analysis between TFs and TCMR/injury specific genes and common genes.

Specific genes associated with different phenotypes were studied in terms of enriched TF motifs using the RcisTarget algorithm. The top three enriched motifs in the different phenotypes are shown in Fig. 4A. Considering that only two specific genes for the ABMR phenotype were found, the RcisTarget algorithm was not used to predict ABMR-related TFs. Histograms of the area under the cumulative recovery curve (AUC) were exhibited, and the AUC scores of motifs that were higher than the mean AUC mean + 3 standard deviations (SDs) were considered as significant motifs (Supplementary Fig. 4A). Motifs with genes recovered scores at > 3 SDs were considered statistically significant (Supplementary Fig. 4B). Our results suggest that common genes in ABMR, TCMR, and injury subtypes might be primarily affected by the transcriptional regulation of RELA (Normalized Enrichment Score (NES) = 9.45, AUC = 0.164) and NFKB1 (NES = 9.35, AUC = 0.162). Similarly, TCMR-specific genes might be mainly regulated by RELA (NES = 10.3, AUC = 0.186), and injury-specific genes might be influenced by the transcriptional regulation of SOX14 (NES = 6.77, AUC = 0.164) (Fig. 4B). Additionally, Pearson’s correlation test between TFs and specific genes was performed to detect regulatory relationships (Fig. 4C). The results showed that RELA correlated significantly negatively with TCMR-specific genes and common genes. NFKB1 and SOX14 correlated positively with common genes and injury-specific genes, respectively. These results suggest that RELA could negatively regulate TCMR-specific genes and common genes. NFKB1 and SOX14 might positively affect ABMR-specific and common genes.

### Identifying hub-specific genes

**Figure 5:**
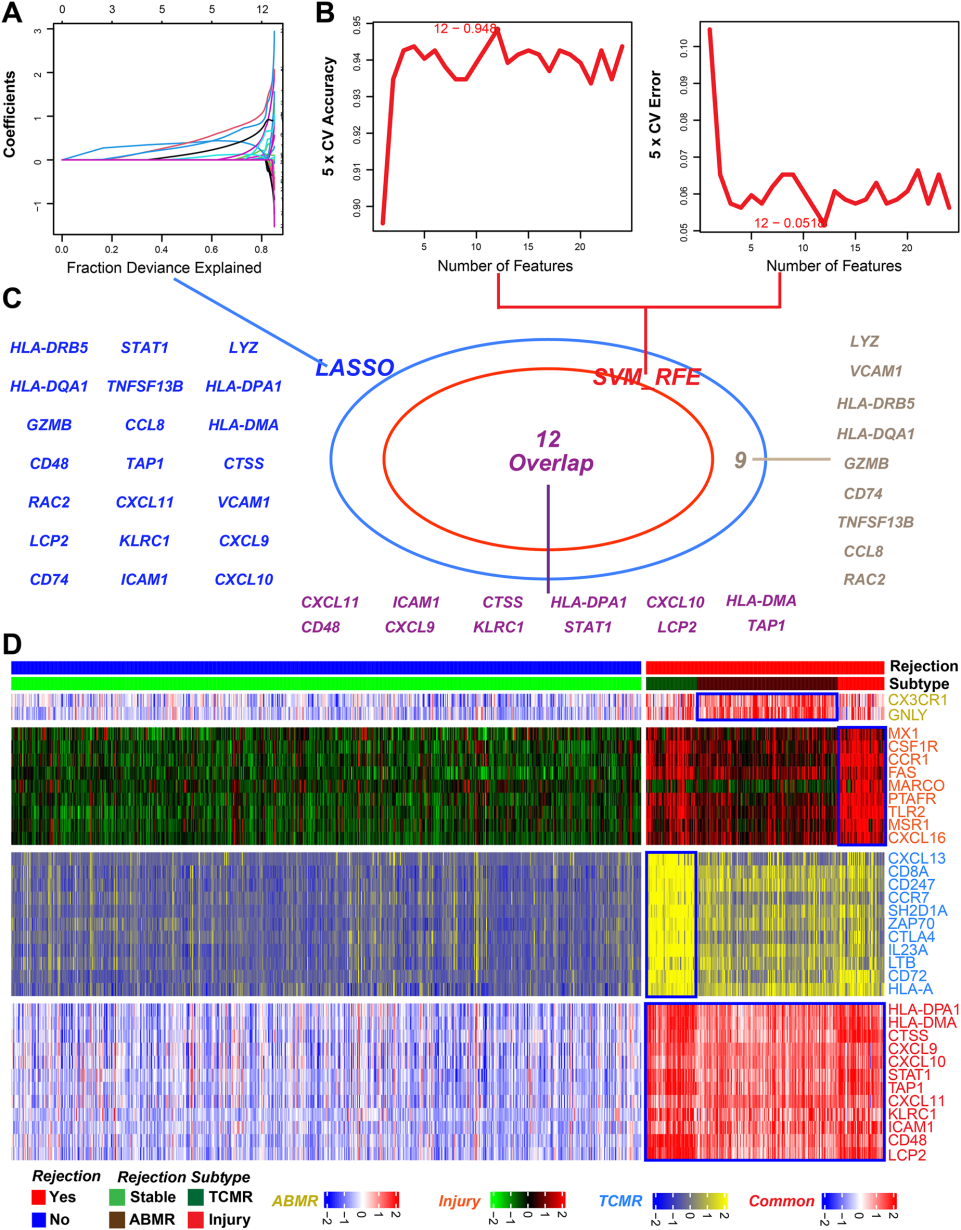
Hub common genes selection using two algorithms. (**A**) LASSO algorithm based on common genes. (**B**) SVM-RFE algorithm based on common genes. (**C**) Take the intersection of the genes obtained by the two algorithms. (**D**) Pheatmap for the hub specific genes expression in different subtypes.

To further narrow the hub-specific genes, the LASSO and SVM-RFE algorithms were run to analyze candidate specific genes. Based on the LASSO algorithm, we selected 21 candidate common genes, 14 candidate TCMR-specific genes, and 12 candidate injury-specific genes (Fig. 5A, Supplementary Fig. 5A,D). Through the SVM-RFE algorithm, we screened out 12 candidate common genes, 16 candidate TCMR-specific genes, and 11 candidate injury-specific genes (Fig. 5B, Supplementary Fig. 5B,E, Supplementary Table 1). Then, common genes identified with the LASSO and SVM-RFE algorithms were identified as hub-specific genes (Fig. 5C, Supplementary Fig. 5C,F). Twelve genes were identified as common hub genes (including CXCL11, ICAM1, and CTSS). Eleven genes were regarded as TCMR-specific hub genes (including CXCL13, CTLA4, and ZAP70), and nine genes were considered as injury-specific hub genes (including CSF1R, TLR2, and FAS). The heatmap for hub-specific genes associated with different phenotypes further verified these results (Fig. 5D).

### Independent verification of hub-specific genes

**Figure 6:**
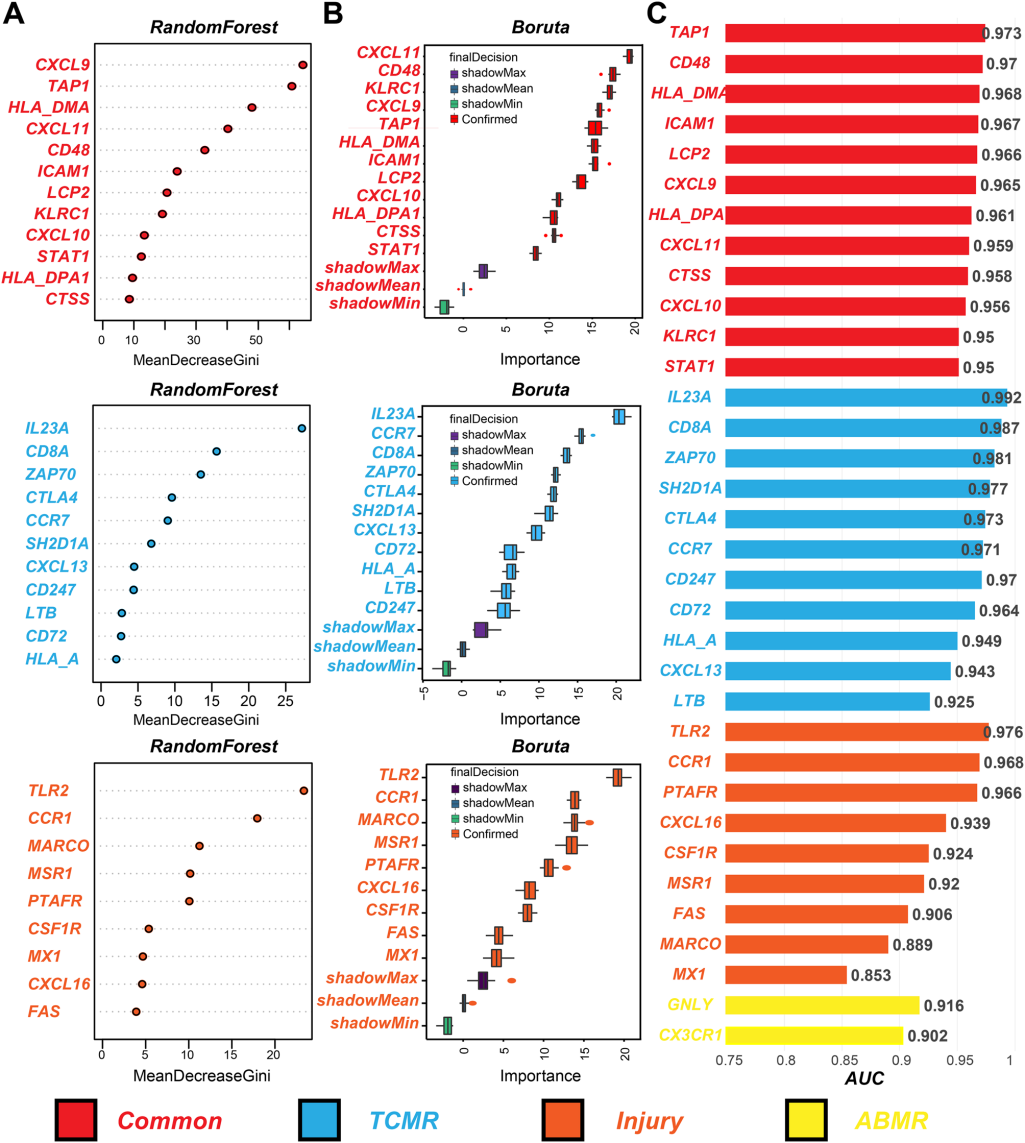
Validation of the hub specific genes using 3 methods. (**A**) Importance ranking of the hub genes in different phenotypes using RandomForest algorithm. (**B**) Features confirmation using Boruta algorithm. (**C**) AUC histogram for the hub specific genes and hub common genes.

We performed three methods to verify the reliability of the selected hub-specific genes. First, the RandomForest algorithm was used to sort the hub-specific genes. CXCL9, TAP1, and HLA-DMA were ranked as the top three common hub genes. We identified IL23A, CD8A, and ZAP70 as the top three TCMR-specific hub genes. In addition, TLR2, CCR1, and MARCO were identified as the top three significant genes (Fig. 6A). Second, the Boruta algorithm was used to confirm all hub-specific genes associated with the different phenotypes (Fig. 6B). Finally, ROC curves revealed that all hub-specific genes had reliable diagnostic utility with the corresponding subtypes (AUC > 0.8) (Fig. 6C).

### Validation of hub-specific genes using an external dataset

**Figure 7:**
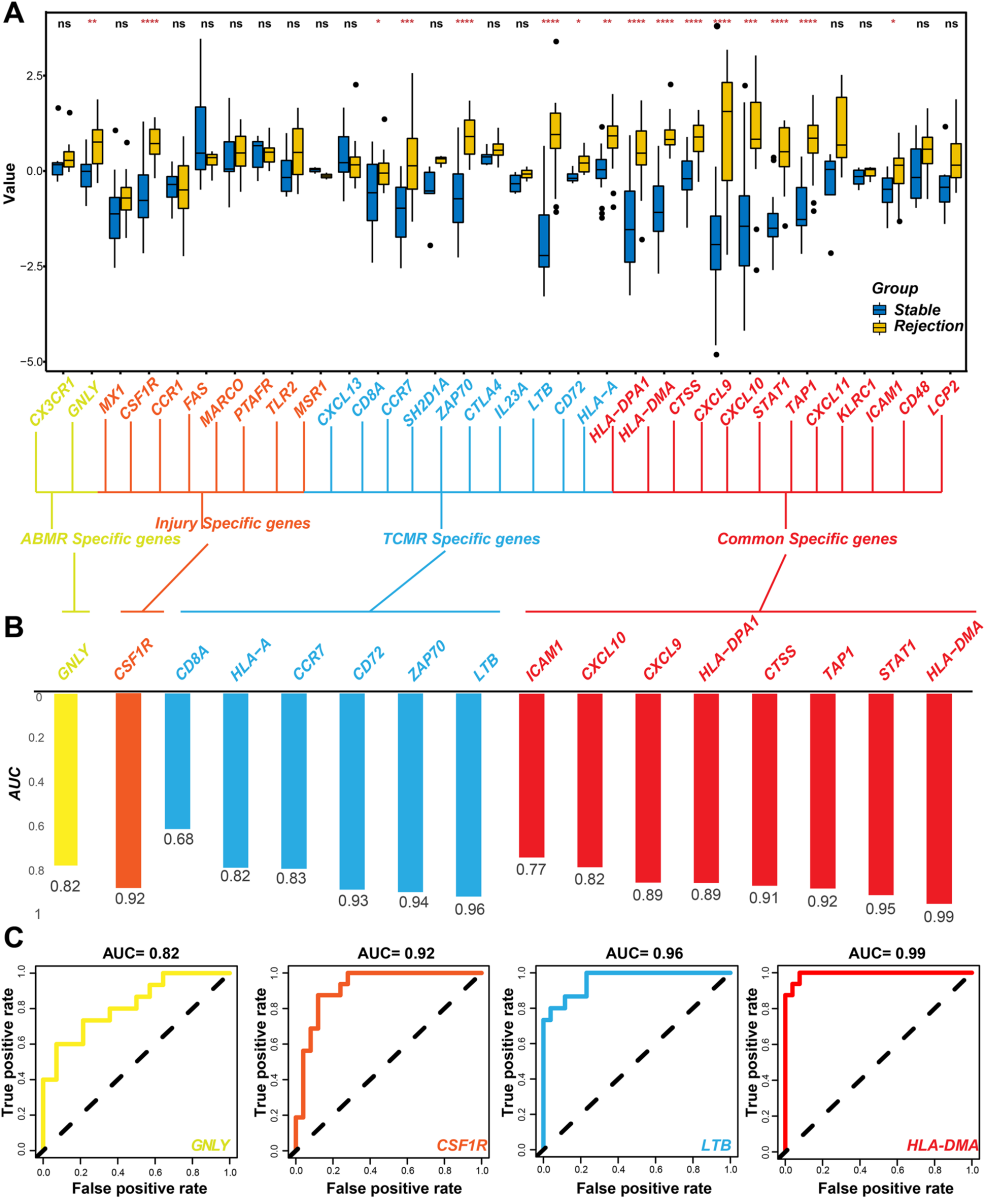
Verification of the hub genes using external test set (GSE2596). (**A**) Differences in hub genes expression between stable and rejection samples (*t* test, *p < 0.05; **p < 0.01; ***, ****p < 0.001). (**B**) AUC histogram for the selected hub genes. (**C**) Part of ROC curves for the selected hub genes.

All hub-specific genes associated with different phenotypes (two ABMR-specific hub genes, eight injury-specific hub genes, 10 TCMR-specific hub genes, and 12 common hub genes) were compared between 27 stable and 16 rejection heart transplant EMB samples. The results indicated that one ABMR-specific hub gene (GNLY), one injury-specific hub gene (CSF1R), six TCMR-specific hub genes (CD8A, HLA-A, CCR7, CD72, ZAP70, and LTB), and eight common genes (ICAM1, CXCL10, CXCL9, HLA-DPA1, CTSS, TAP1, STAT1, and HLA-DMA) were significantly overexpressed in the rejection samples (Fig. 7A). ROC curves suggested that most of the hub-specific genes had a relatively accurate diagnostic capability for rejecting samples (AUC > 0.7) (Fig. 7B). The ROC curves are shown in Fig. 7C.

### Logistic-regression models

**Figure 8:**
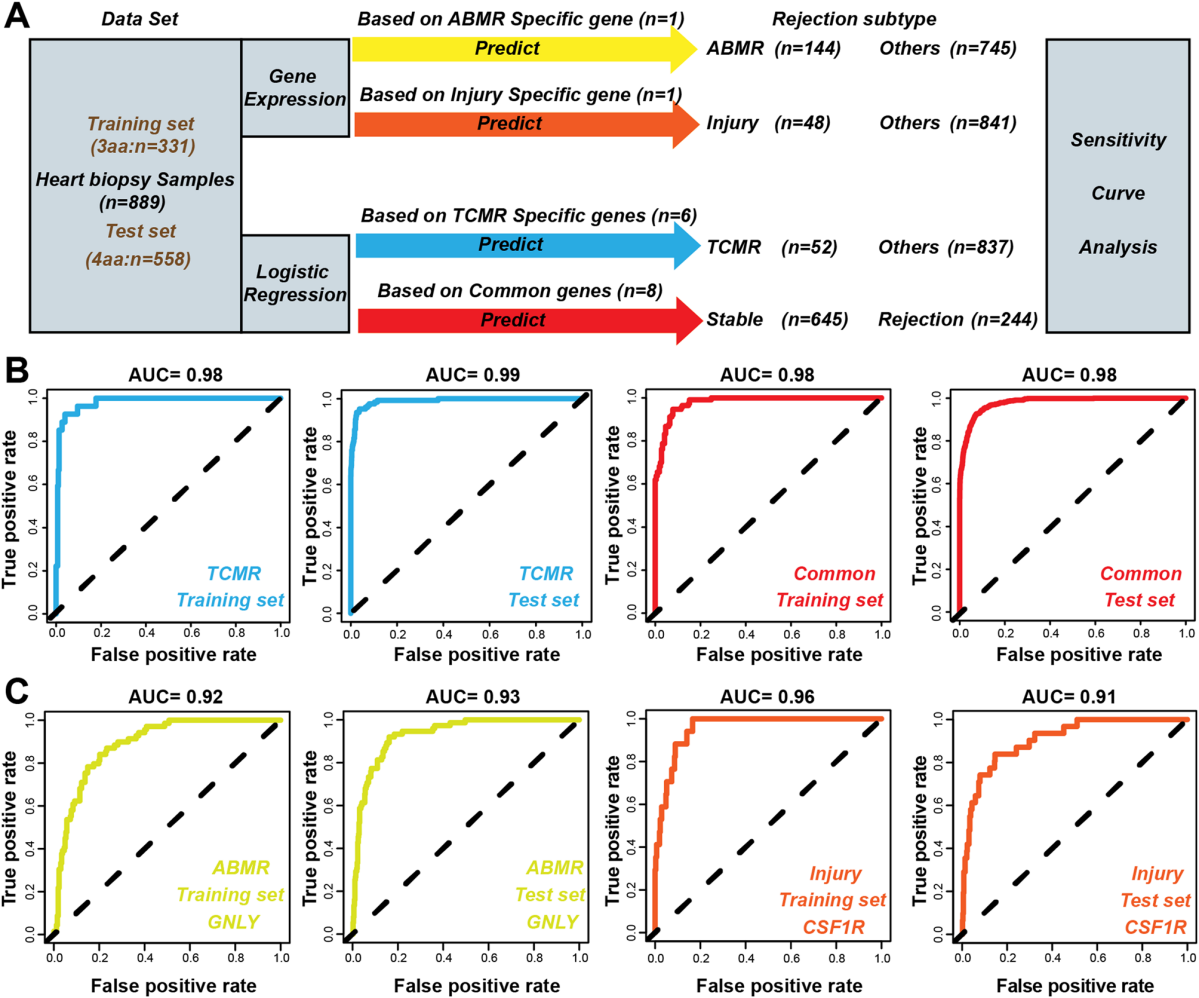
Construction of diagnostic models for different subtypes. (**A**) Workflows for the construction of different models. (**B**) ROC curves for TCMR and common genes logistic regression models. (**C**) ROC curves for ABMR and injury models.

According to Parkes et al., we divided the 889 samples into training and test sets. The initial cohort (331 samples) used to construct the 3AA was regarded as the training set, and the new cohort (558 samples) used to construct the 4AA was considered as the test set. To better diagnose different HTx-rejection phenotypes, logistic-regression models based on six hub TCMR-specific genes or eight common genes were successfully constructed with the training set. ROC curves and AUC scores were performed to evaluate the performance of the models through five-fold cross-validation (Fig. 8A). Surprisingly, we found that logistic-regression models based on six TCMR-specific hub genes or eight common genes could accurately classify TCMR samples (AUC = 0.98), or rejection samples (AUC = 0.98). Moreover, logistic-regression models based on six TCMR-specific hub genes or eight common genes achieved high AUC values (0.99 and 0.98, respectively) with the independent test set (Fig. 8B). Considering that only one ABMR (GNLY) and one injury-specific gene (CSF1R) were successfully validated, the gene-expression level of GNLY or CSF1R was used to distinguish ABMR samples or injury samples from stable samples. ROC curves revealed that GNLY could classify ABMR samples and stable samples well (training set, AUC = 0.89; test set, AUC = 0.93). Similarly, CSF1R also had strong power to classify injury-specific samples and stable samples (training set, AUC = 0.96; test set, AUC = 0.91) (Fig. 8C).

### Gene–drug interactions

**Figure 9:**
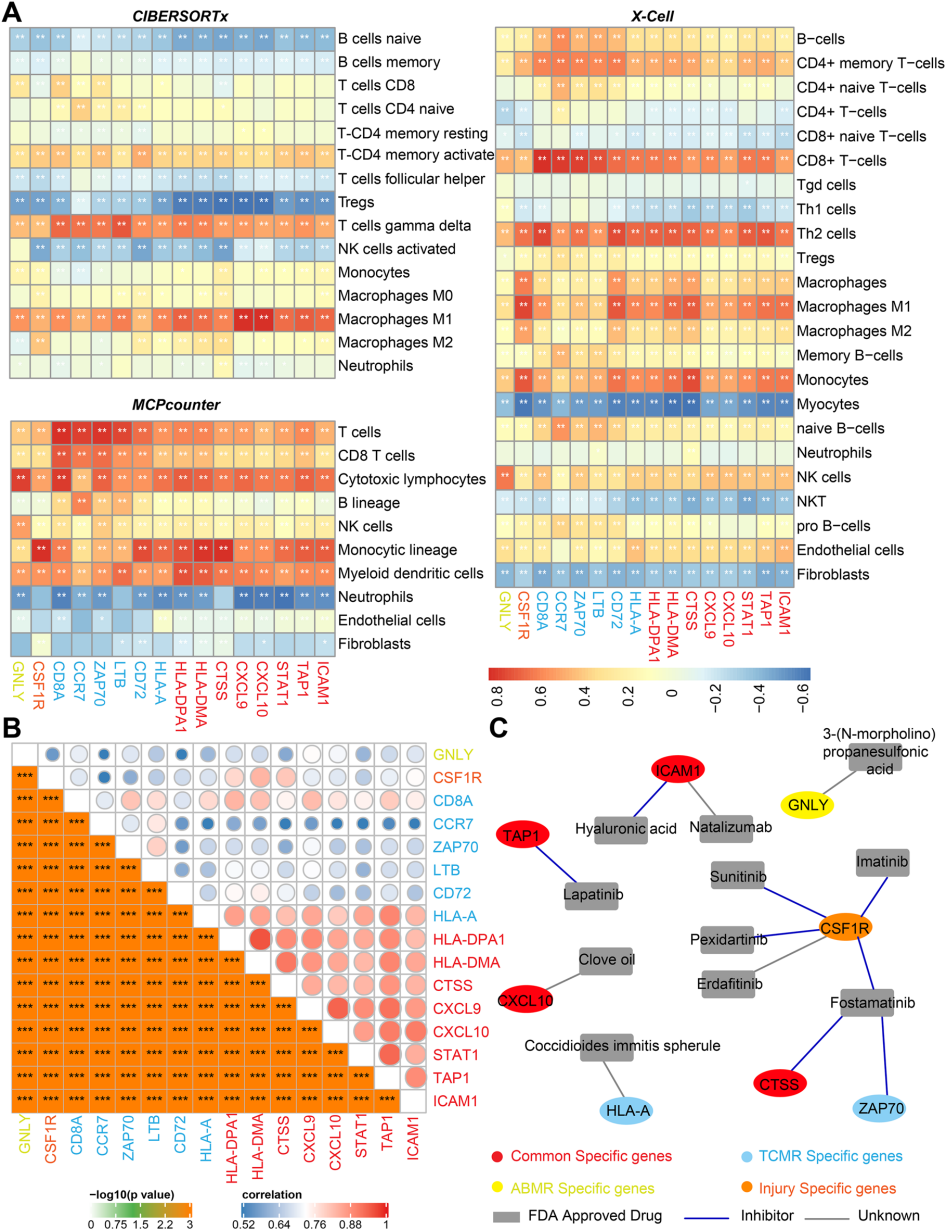
Correlation analysis between the selected hub genes and immune cell infiltration, and drug-gene interaction pairs detection. (**A**) Correlation analysis between the hub specific genes and immune cell infiltration. (**B**) Correlation analysis of different hub specific genes. C. Drug-gene interaction pairs detection using drugbank database (Person correlation analysis was performed, *p < 0.05; **p < 0.01; ***, ****p < 0.001).

To study gene–drug interactions, we first detected correlations between hub-specific genes and immune cell infiltration, based on three different immune-infiltration algorithms. The results showed that these hub-specific genes were significantly correlated with most of the immune cell infiltration, such as T cells, B cells, and macrophages (p < 0.001) (Fig. 9A). Pearson correlation analysis revealed a clear correlation between different hub-specific genes (p < 0.001) (Fig. 9B). Next, all the hub-specific genes were imported into the DrugBank Database to predict the corresponding FDA-approved drugs. Finally, 13 gene–drug pairs were identified, including eight genes (one ABMR-specific hub gene: GNLY; one Injury-specific hub gene: CSF1R; two TCMR-specific hub genes: ZAP70 and HLA-A; four common genes: ICAM1, TAP1, CXCL10, and CTSS), and 11 drugs (Fig. 9C). GNLY can be targeted by 3-(*N*-morpholino) propanesulfonic acid; CSF1R can be inhibited by sunitinib, imatinib, pexidartinib, and fostamatinib; and ICAM1 can be inhibited by hyaluronic acid. In addition, TAP1 can be inhibited by lapatinib, CTSS and ZAP70 can be inhibited by fostamatinib, CXCL10 can be targeted by clove oil, and HLA-A can be targeted by *Coccidioides* *immitis* spherules.

## Discussion

In this study, we employed rigorous bioinformatics screening methods to identify specific genes associated with different subtypes of cardiac transplant rejection. One ABMR-specific hub gene, one injury-specific hub gene, six TCMR-specific hub genes, and eight common hub genes were successfully identified. These key genes exhibited sensitive and specific diagnostic capabilities for different subtypes of cardiac transplant rejection. Interestingly, the majority of these core genes can be targeted by tyrosine kinase inhibitors, suggesting that tyrosine kinases inhibitors may be a promising therapeutic approach for cardiac transplant rejection. Overall, our study provides new insights into the diagnosis and treatment of subtypes of cardiac transplantation.

More immune cell infiltration was observed in the ABMR, TCMR, and injury samples than in the stable samples. Compared to TCMR and injury subtypes, ABMR has poorer immune cell infiltration. In contrast, TCMR exhibited the greatest number of immune cell infiltrates. This means that transplantation patients with TCMR subtype may be the most sensitive to immunosuppressive therapy and the exploration of the transplant rejection mechanisms in ABMR patients should focus on non-immune cells. GO and pathway-enrichment analyses indicated that the key immune-related genes in different phenotypes might play an important role in allograft rejection and immune inflammation response. Meanwhile, we found KRAS signaling was significantly up-regulated in TCMR and chemokine- and leukocyte migration-related functions exactly enriched in injury phenotype. KRAS signaling, as the most frequent mutations in human cancer, has been widely studied and reported^34^. Mamatha Bhat et al. found KRAS signaling is significantly upregulated in patients with posttransplant diabetes mellitus^35^. The targeted elimination of Ras homolog gene family member A (RhoA) specifically in macrophages/monocytes could lead to the suppression of fractalkine receptor expression and effectively prevents chronic rejection of mouse cardiac allografts^28^. Hu et al. discovered that KRAS mutation drived cancer cells to evade surveillance by the innate immune system through activating CD47^36^. Conversely, in cardiac transplant rejection, the elimination of donor CD47 expression can alleviate the rejection of vascularized allografts^37^. These results suggested that the KRAS signaling pathway may play a crucial role in cardiac transplant rejection and warrants further investigation.

Further research showed that TCMR-specific and common genes might receive negative transcriptional regulation of RELA. Common genes may also be positively transcriptionally regulated by NFKB1, and SOX14 could regulate injury-specific genes by transcriptional activation. RELA and NFKB1 (Nuclear Factor NF-Kappa-B P50 Subunit) were also known as two subunits for nuclear factor NF-Kappa-B. Previous data confirmed that the activation of delayed xenograft rejection depends on RELA expression in cardiac vascular endothelial cells^38,39^. NFKB1 has been reported to be activated distinctively during liver transplant rejection^40^. SOX14 is primarily associated with embryonic and neuronal development^41,42^, and its relationship with immunity and inflammation has not yet been revealed. These results convinced us that different transcriptional regulatory patterns may lead to different subtypes of graft rejection.

Current monitoring methods for heart transplant rejection, including Allomap assay and EMB, can effectively identify chronic transplant rejection but lack sensitivity in diagnosing acute rejection reactions. However, controlling acute rejection reactions plays a crucial role in early heart function recovery and prognosis for patients. A prospective transcriptome analysis of endomyocardial biopsies revealed that incorporating molecular feature monitoring can better identify heart transplant rejection^43^. Pham et al. found that post-heart transplant gene expression profiles exhibit sensitivity and specificity comparable to pathological biopsies in monitoring transplant rejection reactions^44^. Here, after deep machine-learning analysis and validation with an external validation set, our study successfully identified one ABMR-specific hub gene, one injury-specific hub gene, six TCMR-specific hub genes, and eight common hub genes with high sensitivity and specificity in diagnosing transplant rejection (AUC score > 0.7). Two TCMR-specific hub genes (CD8A, CCR7) have been reported by Halloran^10^, which further confirmed the reliability of the genes we screened. The GNLY protein is present in the cytotoxic granules of cytotoxic T lymphocytes and natural killer cells, with markedly elevated expression during renal transplant rejection and myocardial infarction^45–47^. Colony-stimulating factor 1 receptor (CSF1R) has been correlated with the production, differentiation, and function of macrophages^48,49^. Bézie et al. confirmed that IL34 can regulate macrophage differentiation towards a regulatory phenotype and induce cardiac transplantation tolerance via CSF1R^50^. As important alleles of major histocompatibility antibodies, the compatibilities of HLA-A, HLA-DPA1, and HLA-DMA are crucial for heart transplant survival^51–53^. HLA mismatch increases the severity and frequency of HTx rejection^54^. CD8 antigen (CD8A) is a cell surface glycoprotein found on most cytotoxic T lymphocytes, which mediates effective cell–cell interactions within the immune system^55^. Additionally, ZAP70, LTB, STAT1, ICAM1, CXCL9 and CXCL10 have been found to be significantly upregulated during transplant rejection and could promote and aggravate heart graft rejection^56–59^. Correlation between TAP1^60^, CTSS and graft rejection has not been revealed.

FDA-approved drugs for different subtypes were also explored. The drugs screened in this study, including Fostamatinib, Sunitinib, Imatinib, Pexidartinib, and Lapatinib, all belong to the class of tyrosine kinase inhibitors (TKIs), most of which are used in clinical cancer treatment. Previous studies have clearly documented the cardiotoxicity of Sunitinib^61,62^ and Imatinib^63–65^, while the cardiotoxicity of Pexidartinib^66^ and Lapatinib has been rarely reported. The clinical application of Sunitinib, Imatinib, Pexidartinib and Lapatinib needs to be extremely cautious. Fostamatinib has been found to improve heart transplant rejection by inhibiting the production of graft-specific antibodies^67^, warranting further investigation. In summary, tyrosine kinase inhibitors may be a promising direction for the development of novel drugs for heart transplant rejection. For example, Janus kinase inhibitors have been widely reported for the treatment of allograft rejection in heart transplantation^68–70^. Interestingly, the main active component of clove oil, eugenol, is considered to have anti-inflammatory and antioxidant properties^71^, which may be beneficial for heart health. Further investigation of the role of eugenol in transplant rejection is meaningful.

In conclusion, we screened one ABMR-specific hub gene (GNLY), one injury-specific hub gene (CSF1R), six TCMR-specific hub genes (CD8A, HLA-A, CCR7, CD72, ZAP70, and LTB), and eight common hub genes (ICAM1. CXCXL10, CXCL9, HLA-DPA1, CTSS, TAP1, STAT1, and HLA-DMA). TFs for different phenotypes were successfully identified (RELA for TCMR, RELA and NFKB1 for rejection, and SOX14 for injury). The corresponding drugs approved by the FDA for the different subtypes were predicted and tyrosine kinase inhibitors may be a promising direction for the development of novel drugs for heart transplant rejection. According to our study, cardiac graft rejection subtypes can be accurately diagnosed by detecting subtype-specific gene expression, and then precise treatment or medication can be performed or administered.

Due to the inevitable difficulty of obtaining secondary heart transplant samples or post-transplant EMB samples, a limitation of this study is that the identified hub-specific genes are difficult to validate with human samples. Further cellular, molecular, and animal experiments are required. The 16 specific hub genes identified in this study merit further attention and exploration. Additionally, transplant rejection is influenced by multiple factors, including genetic and environmental factors, and defining transplant rejection subtypes solely based on molecular features is insufficient. Incorporating sufficient clinical data will help address this limitation.

## Materials and methods

### Datasets and preprocessing

The workflow for this study is shown in Supplementary Fig. 1. RNA-sequencing (RNA-seq) data for heart transplant EMBs (GSE124897, 889 samples; GSE2596, 63 samples) were obtained from the GEO (http://www.ncbi.nlm.nih.gov/geo))^72,73^. According to Parkes et al.^72^, the 889 samples in GSE124897 were classified into four groups: including 645 stable, 52 TCMR, 144 ABMR, and 48 injury samples (Fig. 1A). All 889 samples from the GSE124897 dataset were included in the study. For the GSE2596 dataset, only 27 stable and 16 heart transplant- rejection EB samples were included. All transcriptome data were preprocessed using the limma package of R software for normalization^74^.

### Constructing a co-expression network

First, we calculated the gene variances and selected the top 25% variant genes to construct a co-expression network, using the WGCNA package^75^ of R software. After constructing a sample-clustering tree, 10 outliers were eliminated (Supplementary Fig. 2A). Pearson's correlation matrices for all pairwise genes were generated, after which a weighted-adjacency matrix was constructed. Then, a scale-free network was built based on β = 4 (scale-free R^2^ = 0.9) and used to penalize weak correlations and emphasize strong correlations (Fig. 1C). Second, the adjacency matrix was converted to a topology-overlay matrix (TOM), which was used to compute the network connectivities of different genes. Finally, average-linkage hierarchical clustering was conducted according to the TOM-based dissimilarity measure to generate gene modules containing similar expression patterns.

### Acquiring highly expressed genes associated with the ABMR, TCMR and injury phenotypes

To screen out highly expressed genes linked to the ABMR, TCMR and injury phenotypes, the limma R software package was performed to identify differentially expressed genes between the TCMR, ABMR, and injury phenotypes (log2 fold-change [FC] ≥ 1, false-discovery rate [FDR] < 0.05). The ggplot2 R software package was used to generate a volcano map^76^.

### Immunoscape

The CIBERSORTx^77^, MCPcounter^78^, and X-Cell^79^ algorithms were used to compute the degrees of immune cell fractionation for all 889 samples. Then, the pheatmap R software package was used for clustering and to show the immune cell distributions for all four phenotypes. Differences in immune infiltration between each algorithm were compared using a heatmap.

### Acquisition of immune-related candidate TCMR-, ABMR-, injury-specific genes

First, three immune-related gene sets were downloaded from the Immunogenetic-Related Information Source (IRIS) Database (1489 genes), the Immport Database (1793 genes), and the Immunome Database (881 genes). Then, the intersecting genes of turquoise-module genes and highly expressed genes in ABMR/TCMR/injury samples present in the three immune-related gene lists, were considered key genes for the ABMR/TCMR/injury samples. Finally, the intersection between the key ABMR, TCMR, and injury key genes was determined to identify common genes and phenotype-specific genes.

### Biological function and transcription factor-enrichment analysis

Metascape (https://metascape.org/) is a freely available web tool designed to provide experimental biologists with a comprehensive resource for gene-list annotation and analysis^80^. We used Metascape to perform functional and pathway-enrichment analyses for key ABMR/TCMR/injury-related genes. A p value of < 0.01 was considered to reflect a significant difference.

The RcisTarget algorithm^81^ was used to predict the over-representation of TCMR- and injury-specific genes, as well as common genes in transcription factor (TF)-binding motifs (species, Homo sapiens; search space, 500 base pairs upstream of the transcription-start site; number of orthologous species, 10). Gene-motif ranking and motif-to-transcription factor annotation databases were used in this analysis. The motifAnnotations_hgnc tool (version 9; ‘mc9nr’, 24,453 motifs) was used to annotate the transcription factor motifs.

### Selection of hub-specific genes

To narrow down specific genes in different heart transplant-rejection subtypes, two different algorithms were used to select hub-specific genes from candidate specific genes. Penalty parameter adjustment with tenfold cross-validation, based on the least absolute shrinkage and selection operation (LASSO) algorithm, was used to select hub-specific genes^82^. In addition, the Support Vector Machine-Recursive Feature Elimination (SVM-RFE) algorithm was used to identify hub-specific genes^83^. Finally, hub-specific genes were defined after combined analysis with the LASSO and SVM-RFE algorithms.

### Validation of hub-specific genes

After identifying hub-specific genes, three algorithms were used to assess their relative importance for the different heart transplant-rejection phenotypes. The RandomForest^84^ and Boruta algorithms^85^ were used to sort hub-specific genes by importance. ROC curves were used to demonstrate the utility of hub-specific genes to diagnose different subtypes of heart transplant rejection. Additionally, all hub gene-expression differences between the stable and rejection samples were analyzed with an external test set (GEO accession number GSE2596). Similarly, ROC curves were generated to assess the diagnostic utility of hub-specific genes for rejection samples (GEO accession number GSE2596).

### Constructing logistic-regression models

After validating the hub-specific genes, one ABMR, one injury, and six TCMR hub-specific genes, as well as eight common genes were chosen for further analysis. We constructed two logistic-regression models based on six TCMR-specific hub genes and eight common hub genes for diagnosing TCMR or rejection samples. Considering that only one ABMR- and one injury-specific hub gene were successfully validated, we used two gene-expression levels for diagnosing ABMR or injury. ROC curves were mapped to measure the diagnostic utility of the logistic-regression models and ABMR/injury-specific genes.

### Predicting gene–drug interactions

DrugBank (https://www.drugbank.com/) is a web-based database containing information on a wide range of drugs and their interactions with targets. Successfully validated hub-specific genes were imported into the DrugBank Database to identify their targeted drugs. Only FDA-approved drugs were regarded as reliable targeted drugs. Then, a network of interactions between hub-specific genes and drugs were constructed using Cytoscape software (version 3.7.1)^86^.

### Statistical analysis

All images are generated using R software (version 4.1.0). The *t*-test was applied for comparisons between two groups. The correlations between the two groups were calculated and assess by Pearson correlation test. The glmnet and e1071 packages of R software (version 4.1.0.) were used to perform the LASSO and SVM-RFE algorithms, respectively. Statistical significance was set at a threshold of p < 0.05.

### Supplementary Information

Supplementary Information.

## Data Availability

The datasets analyzed for this study can be found in the Gene Expression Omnibus (https://www.ncbi.nlm.nih.gov/geo/, GSE124897, GSE2596).
