# Supplementary material for: Exploring personalized treatment for cardiac graft rejection based on a four-archetype analysis model and bioinformatics analysis
Source: Sci Rep. 2024 Mar 19;14:6529. doi: 10.1038/s41598-024-57097-9 (PMC10948767; doi:10.1038/s41598-024-57097-9)
Supplement: Supplementary file 1 — Supplementary Information. [file 41598_2024_57097_MOESM1_ESM.pdf]

Supplementary Figure

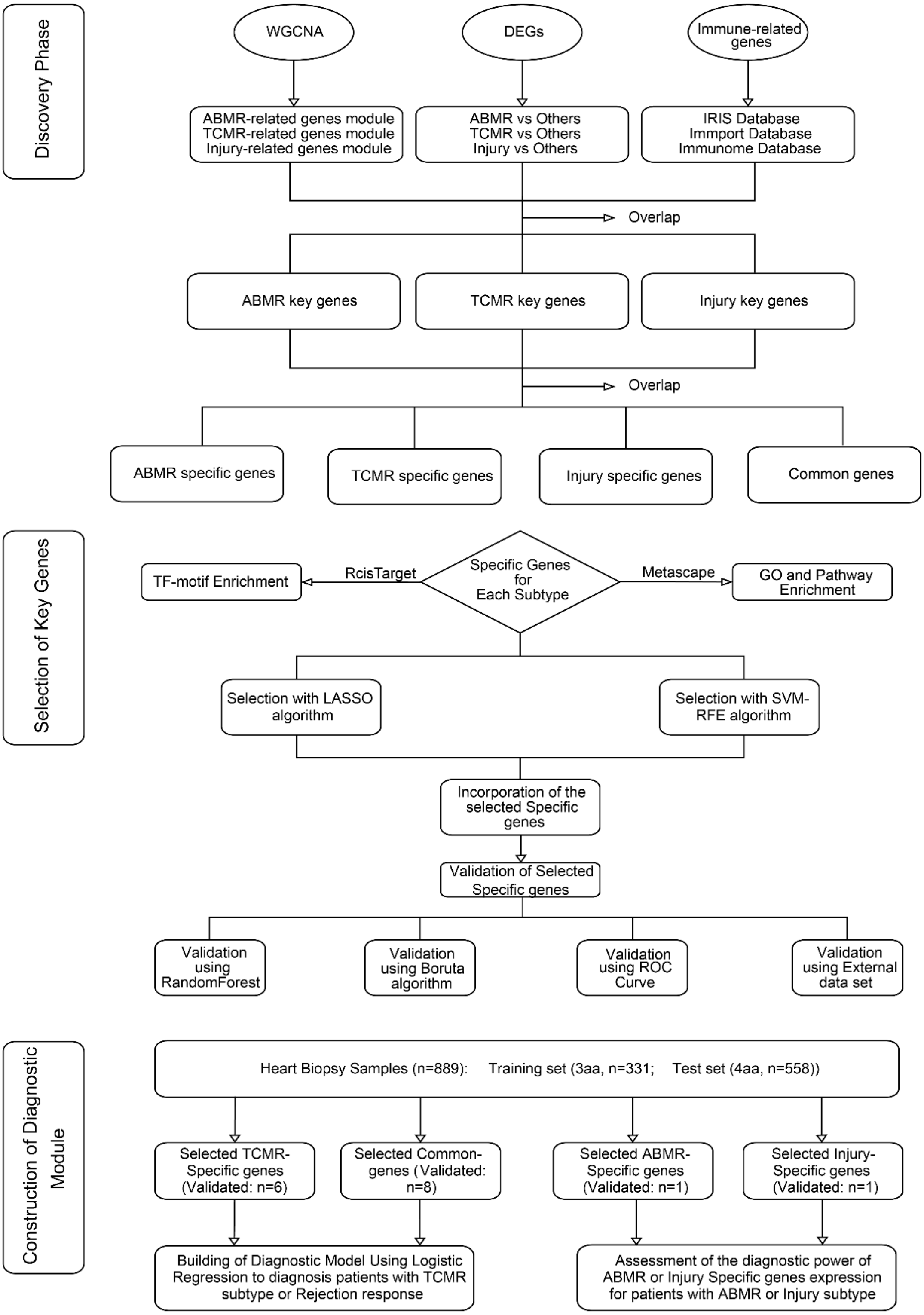

Supplementary Fig. 1. The flow chart of the study.

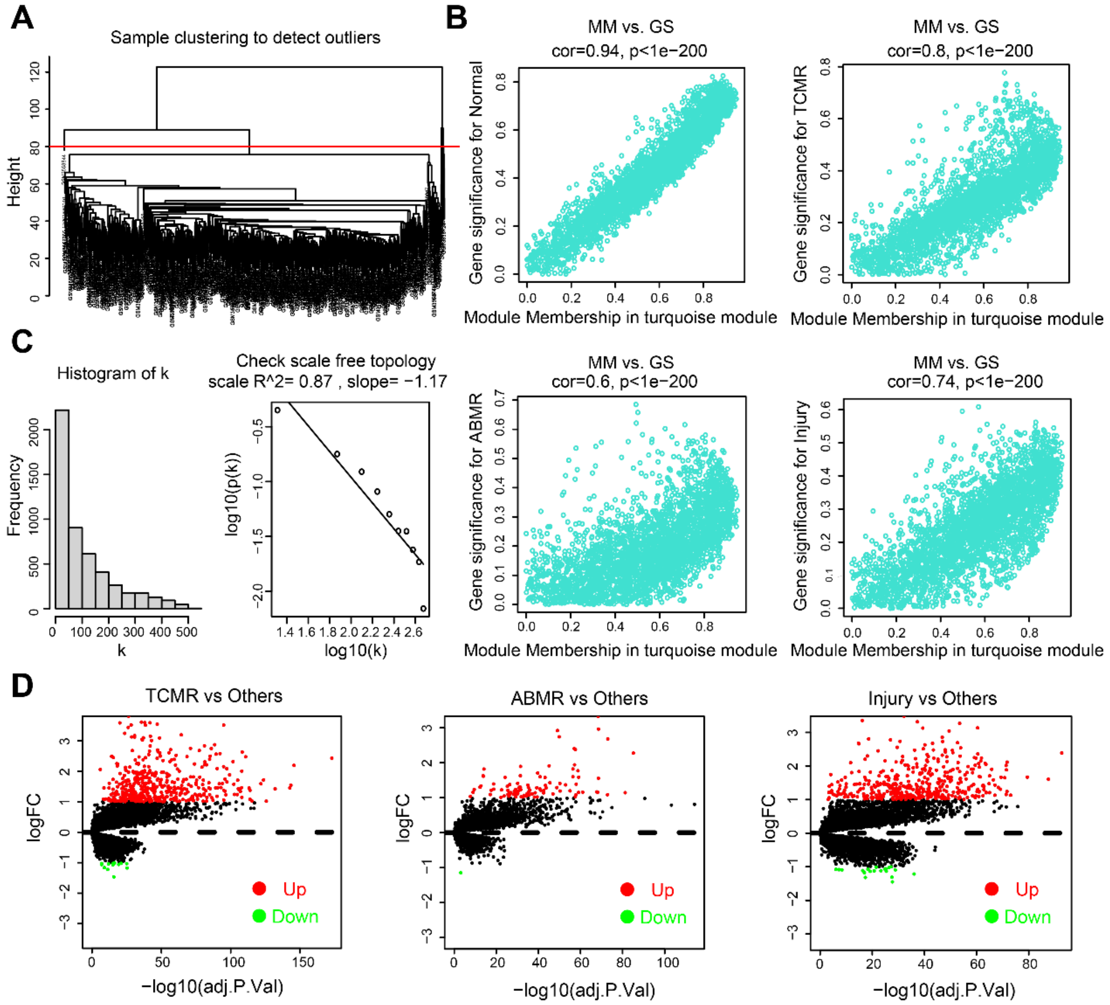

Supplementary Fig. 2. Construction of co-expression network and differential genes analysis between ABMR/TCMR/injury phenotypes and others. A. Sample clustering tree in GSE124897. B. Correlation analysis between turquoise module and 4 phenotypes. C. Scale free topology histogram when soft-thresholding power β = 4. D. Volcano maps for the differential genes between ABMR/TCMR/injury phenotypes and others.

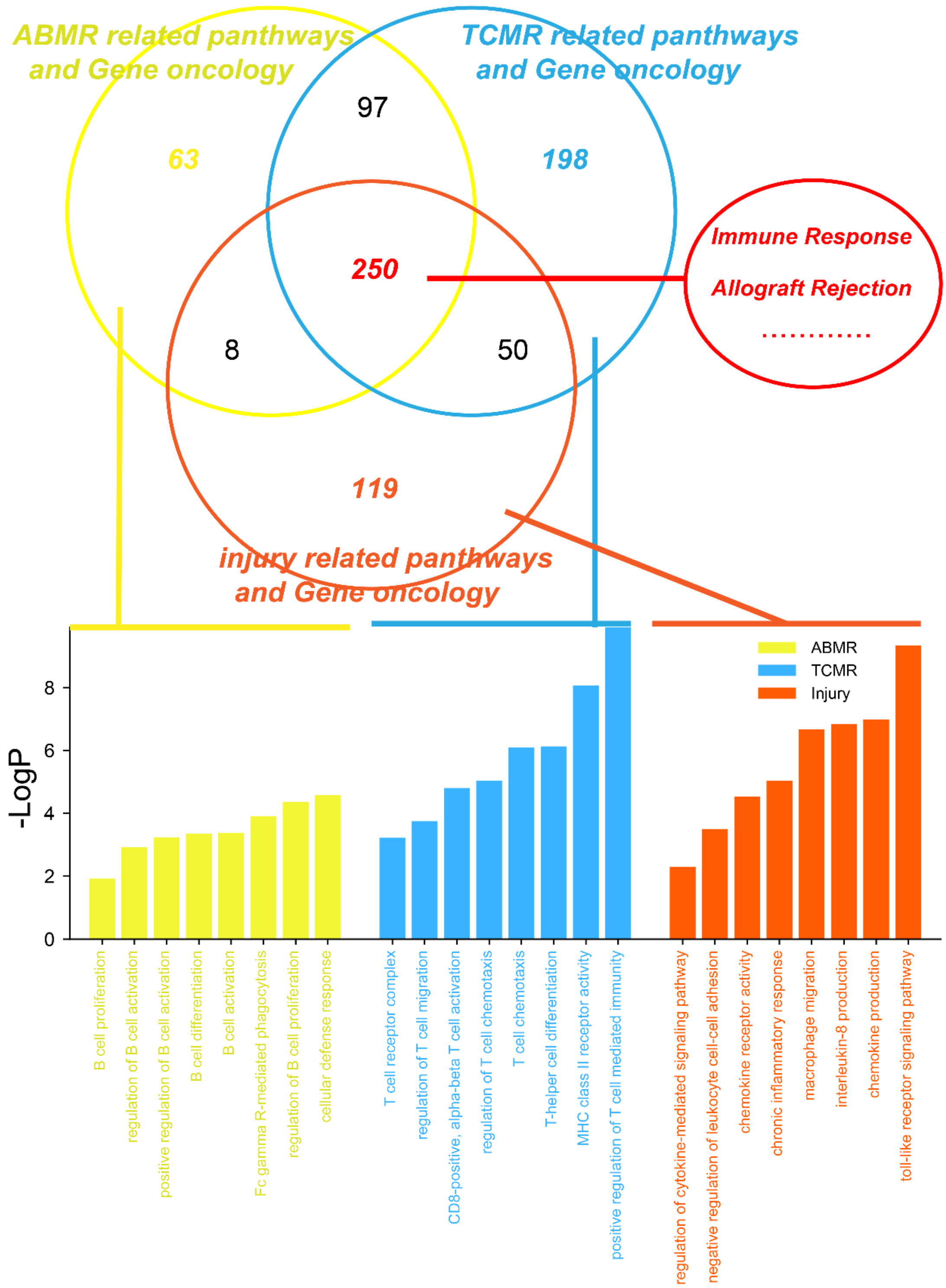

Supplementary Fig. 3. Gene oncology and pathway enrichment analysis for different phenotypes.

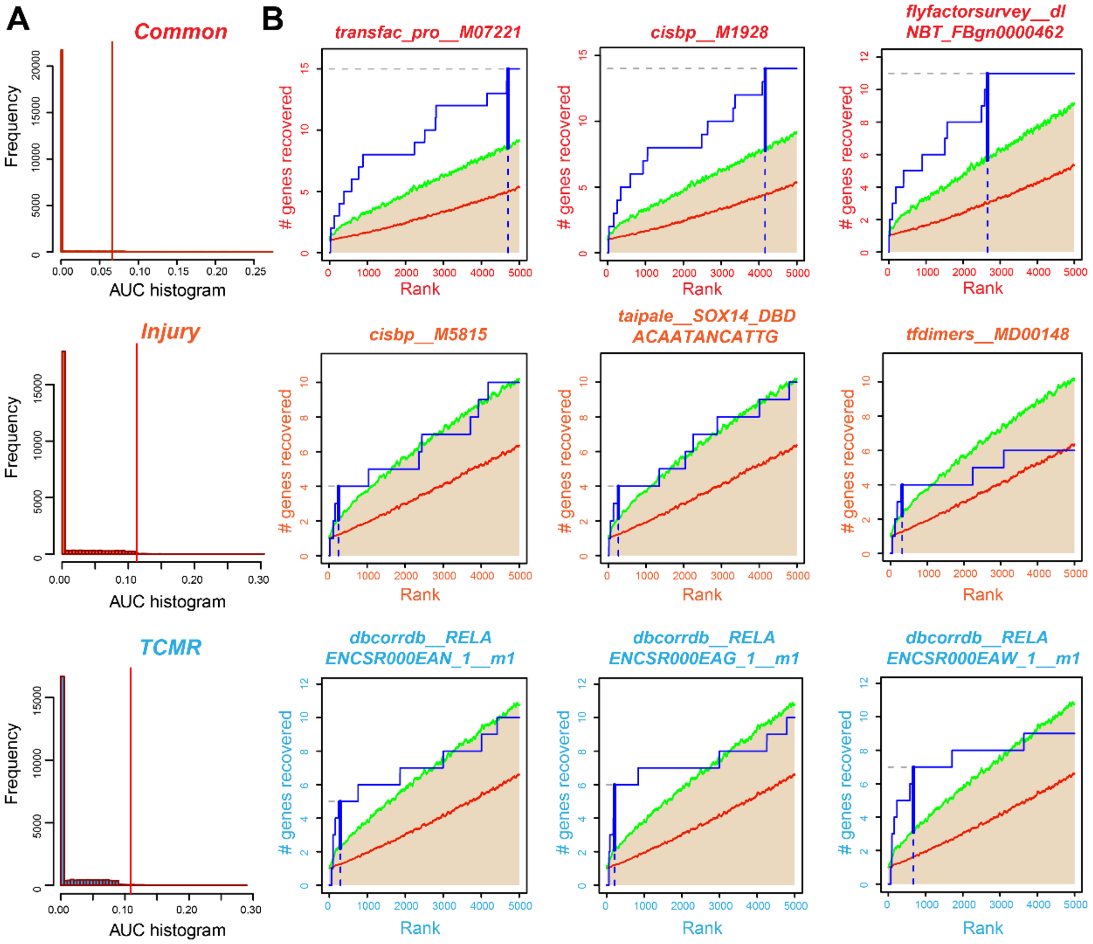

Supplementary Fig. 4. Histogram of AUC and selection of significant motifs. A. Histogram of the Area under the cumulative recovery curve. The red vertical line demonstrates the significance level that motifs with a AUC greater than the significance level (AUC mean+3 SD) are considered significant motifs. B. Cumulative recovery curve for top 3 motifs in different phenotypes. The red line represents the global mean of the number of genes recovered and the green line represents 3SD. Motifs greater than 3SD are identified statistically significant.

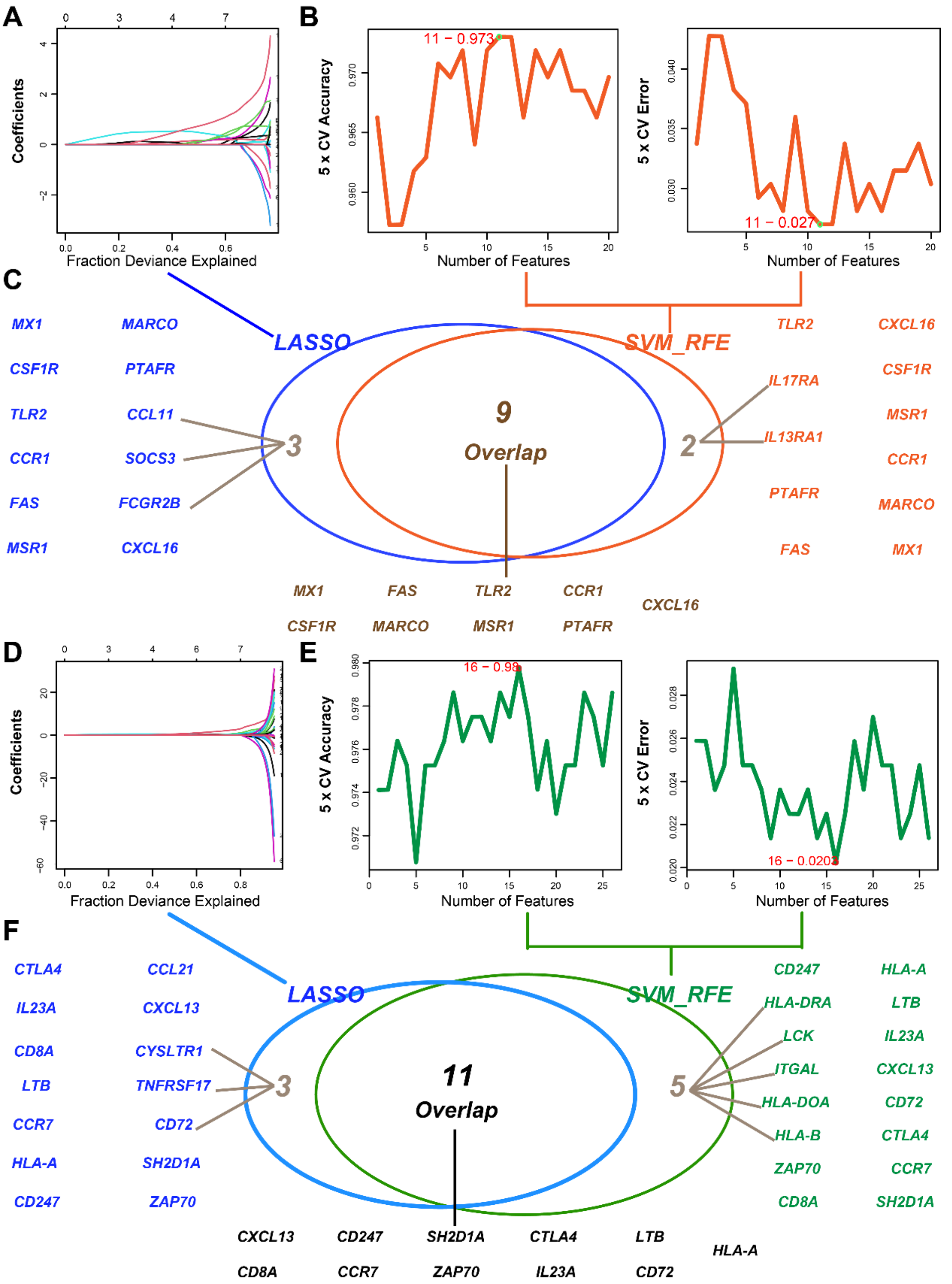

Supplementary Fig. 5. Hub TCMR/injury genes selection using two algorithms. A and D. LASSO algorithm based on specific genes. B and E. SVM-RFE algorithm based on specific genes. C and F. Take the intersection of the genes obtained by the two algorithms.

Supplementary Table1. Average ranking (AvgRank) order based on the 10 folds cross validation. The lower the avgrank, the more important the feature is.

FeatureName	AvgRank	Phenotypes
CXCL11	1.4	Common genes
CD48	2.2	Common genes
ICAM1	7	Common genes
CXCL9	8	Common genes
CTSS	8.4	Common genes
KLRC1	8.4	Common genes
HLA_DPA1	8.6	Common genes
STAT1	9.4	Common genes
CXCL10	9.8	Common genes
LCP2	10	Common genes
HLA_DMA	11	Common genes
TAP1	11.4	Common genes
CD74	12.2	Common genes
RAC2	14.8	Common genes
LYZ	15.6	Common genes
HLA_DQA1	16	Common genes
PTPRC	16	Common genes
VCAM1	16.8	Common genes
HLA_DRB5	16.8	Common genes
CCL8	17	Common genes
GZMB	19	Common genes
FCGR3A	19.2	Common genes
TNFSF13B	19.4	Common genes
ITGB2	21.6	Common genes
TLR2	1	injury specific genes
MSR1	3.8	injury specific genes
MX1	4	injury specific genes
PTAFR	4	injury specific genes
FAS	5	injury specific genes
CXCL16	5.4	injury specific genes
CSF1R	7.6	injury specific genes
IL17RA	9.6	injury specific genes
CCR1	11.4	injury specific genes
MARCO	11.4	injury specific genes
IL13RA1	12	injury specific genes
FCGR2B	12.8	injury specific genes
S100A8	13.4	injury specific genes
TLR4	14	injury specific genes
CCL11	14.2	injury specific genes
CXCL2	14.8	injury specific genes

BTK	15	injury specific genes
C5AR1	16.2	injury specific genes
CCL3	17	injury specific genes
SOCS3	17.4	injury specific genes
CD247	3.4	TCMR specific genes
HLA_DRA	4.4	TCMR specific genes
CCR7	5.2	TCMR specific genes
SH2D1A	6	TCMR specific genes
CD72	6.2	TCMR specific genes
IL23A	7.8	TCMR specific genes
ZAP70	8.2	TCMR specific genes
CD8A	9	TCMR specific genes
HLA_A	10	TCMR specific genes
LTB	10.4	TCMR specific genes
HLA_B	12.8	TCMR specific genes
CXCL13	13.2	TCMR specific genes
ITGAL	13.2	TCMR specific genes
CTLA4	13.6	TCMR specific genes
HLA_DOA	15.8	TCMR specific genes
LCK	15.8	TCMR specific genes
CYSLTR1	16.2	TCMR specific genes
CCL21	17.8	TCMR specific genes
HLA_E	17.8	TCMR specific genes
TNFRSF17	18.6	TCMR specific genes
IL2RG	19	TCMR specific genes
IL7R	20.4	TCMR specific genes
IFNG	21	TCMR specific genes
TNFRSF9	21.4	TCMR specific genes
ICOS	21.8	TCMR specific genes
IL32	22	TCMR specific genes
